# Psychosis Mental Health Campaigns: A Scoping Review of Strategies and Outcomes

**DOI:** 10.1111/eip.70206

**Published:** 2026-07-10

**Authors:** Kelsey T. Straub, Kara Pemberton, Mackenzie Tennison, Sydney McKinstry, Sarah Kopelovich

**Affiliations:** ^1^ Department of Psychiatry & Behavioral Sciences University of Washington School of Medicine Seattle WA USA

**Keywords:** early medical intervention, health communication, help‐seeking behaviour, mental health, psychotic disorders, public health, scoping review

## Abstract

**Objective:**

Early intervention in psychosis is associated with improved outcomes, yet prolonged duration of untreated psychosis remains a major barrier globally. Public health campaigns offer a promising strategy for promoting early detection and help‐seeking, although findings on their effectiveness remain mixed. This scoping review examines the characteristics, strategies, and outcomes of psychosis public health campaigns published between 2008 and 2025, with particular attention to metrics that are used to evaluate campaign success.

**Methods:**

Six databases were searched for peer‐reviewed studies reporting outcomes of psychosis public health campaigns. Studies were included if they reported on campaign effectiveness and used a comparison condition. Data extraction focused on campaign characteristics, strategies, messages, target audiences, and outcomes.

**Results:**

Nineteen studies describing 14 distinct campaigns met inclusion criteria. Campaigns varied widely in geographic scope, target audiences, duration, and messaging strategies. There was substantial heterogeneity in how duration of untreated psychosis was defined. Although some campaigns demonstrated significant reductions in overall duration of untreated psychosis, most showed mixed effects or improvements in subcomponents only. Campaigns employing multi‐modal and sustained engagement strategies were more likely to report positive outcomes. Recent digital campaigns offered targeted outreach but showed inconsistent effects.

**Conclusion:**

Findings highlight the need for standardized definitions of the duration of untreated psychosis construct and consideration of more proximal outcomes for a public health intervention. Future campaigns should incorporate digital strategies, stakeholder‐informed messaging, and alternative success metrics—such as referral volume and service engagement—to more accurately capture impact and guide implementation efforts in diverse settings.

**Abbreviations**
CALM: Clear Answers to Louisiana Mental Health
CHR‐P: Clinical high risk for psychosis
CIEIS: Camden and Islington Early Intervention Service
CORS: Circumstances of Onset and Relapse Schedule
DETECT: Dublin East Treatment and Early Care Team
DUP: Ad.Med; Time from psychosis onset to adequate medication treatment, defined as 30 consecutive days of antipsychotic treatment unless positive symptoms remit sooner
DUP: Duration of untreated psychosis
DUP‐Any: Time from psychosis onset to initiation of any treatment for psychosis
DUP‐EIS: Time from psychosis onset to enrollment in early intervention services
DUP‐Help: Time from psychosis onset to first help‐seeking contact
DUP‐Med: Time from psychosis onset to initiation of medication treatment (also referred to as DUP‐Demand by some campaigns)
DUP‐Refer: Time from first help‐seeking contact to enrollment in early intervention services
DUP‐Service: Time from psychosis onset to referral to early intervention services
DUP‐Supply: Time from initiation of medication treatment to enrollment in early intervention services
EASY: Early Assessment Service for Young People with Psychosis
EIS: Early intervention services
FETZ: Cologne Early Detection & Therapy Center for Mental Crises
IRAOS: Interview for the Retrospective Assessment of the Onset of Schizophrenia
JBI: Joanna Briggs Institute
LEGs: Liaison and Education in General Practices
PANSS: Positive and Negative Syndrome Scale
PEPP: Prevention and Early Intervention in Psychosis Program
PRISMA‐ScR: Preferred Reporting Items for Systematic Reviews and Meta‐Analyses Scoping Review
REDIRECT: BiRmingham Early Detection In untREated PsyChosis Trial
SEO: Search engine optimization
SIPS: Structured Interview for Psychosis‐risk Syndromes
TIPS: Treatment and Intervention in Psychosis

## 1 Introduction

Untreated or poorly managed psychotic disorders are associated with poor health, functional, and quality of life outcomes (Alonso et al. 2018; Charlson et al. 2018; Kessler et al. 2005; Solmi et al. 2023). However, reducing the duration of untreated psychosis (DUP)—the delay from psychosis onset to treatment initiation—beneficially alters the psychosis trajectory and has a sustained impact which can be seen decades later in reduced symptomatology, improved functioning, and improved quality of life (Cechnicki et al. 2014; Howes et al. 2021; O'Keeffe et al. 2022; Penttilä et al. 2014; Starzer et al. 2023).

Nearly 20 years ago, in an effort to improve the long‐term prognosis of psychotic disorders, the World Health Organization set a target to reduce average DUP to less than 3 months (Bertolote and McGorry 2005), spurring international investment in early intervention services (EIS) for psychosis (George et al. 2022; Heinssen and Azrin 2022). Despite these investments, DUP remains high throughout much of the world, including many US communities (Salazar De Pablo et al. 2024). Thus, it has become evident that increasing availability of eEIS is typically insufficient to reduce DUP. Reducing DUP is a complex target likely requiring a multi‐faceted strategy. One such strategy which has achieved varying degrees of success is conducting a public health campaign aimed at increasing awareness of the signs of psychosis and available treatment resources (Lloyd‐Evans et al. 2011; Murden et al. 2024).

Understanding why some public health campaigns have been successful in reducing DUP while others have not requires a nuanced examination of the drivers of DUP. One model conceptualizes DUP as comprising two subcomponents (e.g., Malla et al. 2021). Delays in help‐seeking (DUP‐Help; from psychosis onset to first help‐seeking contact) are often driven by patient‐level characteristics, such as age, premorbid adjustment, symptomatology, stigma, and knowledge of psychosis or psychiatric concerns (Bay et al. 2016; Bechard‐Evans et al. 2007; Malla et al. 2021). Delays in referral (DUP‐Refer; from first help‐seeking contact to enrollment in EIS) tends to be driven by characteristics of the systemic care pathways such as previous contact with the healthcare system, type of first help‐seeking contact, and referrers' knowledge of early psychosis warning signs and treatment resources (Bay et al. 2016; Bechard‐Evans et al. 2007; Birchwood et al. 2013; Malla et al. 2021). Strategies that target the drivers of DUP‐Help and DUP‐Refer are needed to help bring DUP into the prescribed 3‐month window.

Despite the promise of public health campaigns as a strategy to reduce DUP, relatively little published research has directly compared these campaigns or synthesized evidence on their effectiveness across studies. Several reviews have documented efforts to reduce DUP and acknowledge that public health campaigns hold potential in this area. However, many such campaigns have not demonstrated consistent or measurable effects (Lloyd‐Evans et al. 2011; Murden et al. 2024; Oliver et al. 2018). One challenge in drawing firm conclusions is the variation in how DUP is defined, measured, and analysed across studies. Inconsistent findings may also reflect heterogeneity in campaign strategies, implementation approaches, target populations, timing, and external contextual factors such as local policies or political climates. Existing reviews have not reached consensus on which elements contribute to the success of a public health campaign, and few provide sufficient detail about campaign components to allow for a meaningful evaluation of their effectiveness. This lack of specificity limits the field's ability to derive actionable insights or practical guidance for designing future campaigns. Furthermore, DUP is a rather distal metric of campaign effectiveness; campaigns that failed to reduce DUP may still have affected other metrics, such as EIS enrollment, which not only give important context to interpreting DUP results but also serve as important targets in and of themselves. Given that public health campaigns are often resource‐intensive and time‐consuming, a more nuanced understanding of what makes them effective is essential to ensure strategic investment and impact.

The current review builds upon previous research by identifying how DUP has been operationalized across public health campaign investigations and by cataloguing additional outcomes used to evaluate their effectiveness. By including studies that report either DUP (however defined) or relevant proxy outcomes, we aim to capture a more holistic understanding of campaign impact along the early detection pathway. This broader inclusion allows for a more comprehensive assessment of campaign performance metrics and facilitates insights into the mechanisms through which campaigns may influence access to care, even when direct reductions in DUP are not observed. In light of the rapid expansion of digital and social media platforms that have transformed public health campaign strategies (Guo et al. 2020; Wang et al. 2023), this review focused on studies published from 2008 onward. Collectively, this scoping review synthesizes details of campaign design, strategies, and outcomes to distill best practices that can inform the next generation of psychosis awareness campaigns.

## 2 Method

### 2.1 Approach

This scoping review was conducted using the methodological approach outlined by the Joanna Briggs Institute (JBI; Joanna Briggs Institute 2020) and is reported in accordance with the Preferred Reporting Items for Systematic Reviews and Meta‐Analyses Scoping Review (PRISMA‐ScR; Tricco et al. 2018) recommendations. No protocol was registered for this review. The completed PRISMA‐ScR checklist is available in the supplement.

### 2.2 Research Questions

The objective of this review is to identify and synthesize common objectives, strategies, targeted audiences, and outcomes of psychosis public health campaigns published between 2008 and 2025. In line with this aim, studies were only included if they reported metrics related to campaign outcomes; studies describing campaigns with no evaluation of relevant outcomes were not included in the current review.

Within these parameters, our review was guided by the following research questions: What public health campaigns have been conducted for psychosis? Where were these campaigns conducted? What audiences were targeted? What were the campaign strategies deployed? What outcomes have been examined that could serve as proxies for DUP (i.e., other outcomes relevant to facilitating treatment access, such as referrals to EIS)? Which strategies demonstrated effectiveness in reducing DUP or affecting DUP proxy outcomes?

### 2.3 Eligibility Criteria

In line with JBI recommendations, eligibility criteria were based on the type of participants included in the study, the concepts examined in the study, the context in which the study was conducted, and the types of evidence sources comprising the study.

#### 2.3.1 Participants

Studies examining psychosis public health campaigns were included regardless of study participants.

#### 2.3.2 Concept

Psychosis public health campaigns were defined as organized initiatives designed to increase awareness of psychosis and/or promote help seeking among the public or key referral sources. In accordance with the review's focus on evaluating the effectiveness of campaign strategies in promoting access to treatment and reducing treatment delay, studies were included if they reported a quantitative analysis testing the impact of a campaign on either (a) DUP, as defined by the study, or (b) a conceptually related outcome reflecting earlier detection or engagement with care (e.g., changes in referral rates, help‐seeking behaviours, or EIS enrollment). Eligible studies were required to include a comparison condition, such as pre‐ versus post‐campaign data, or a contemporaneous control group, to permit assessment of campaign impact.

#### 2.3.3 Context

Studies conducted in all settings and geographic locations were included.

#### 2.3.4 Source

This review included original peer‐reviewed research studies published between 2008 and 2025. Meta‐analyses, systematic reviews, trial protocols for studies that had not yet been conducted, and grey literature were excluded. Publications in all languages were screened with use of Google Translate to determine study acceptability.

### 2.4 Search Strategy

The search strategy was developed by our research group in consultation with a university librarian. Searches were conducted using PubMED, CINAHL Complete, APA PsycArticles, Communication Source, Global Health, and Web of Science. We used multiple terms related to psychosis (“psychosis”, “psychotic disorders”, or “schizophrenia”), early detection (“intervention”, “detection”, “assessment”, or “screening”), and public health campaigns (“campaign” or “health communication”). Relevant article reference lists were also referenced for additional qualifying articles. The initial search was conducted on September 15, 2023 and updated on March 27, 2025. See Supporting Information for full search strategy.

### 2.5 Study Selection

Articles were imported into Covidence (Veritas Health Innovation 2024), a service designed for systematic and scoping review analyses. Duplicate studies were removed; title and abstract screening, full text review, and data extraction were all completed in Covidence. In line with PRISMA standards, the selection process comprised two stages: a title and abstract review followed by a full text review. Two reviewers independently screened the title and abstract of articles to determine if they met the eligibility criteria. Studies were excluded if they did not meet inclusion criteria. Those with questionable qualifications were escalated to full text review. Disagreements were resolved via consensus and were moved to full text review if ambiguity remained. Agreement by two independent reviewers was required for full text reviews. Disagreements were resolved via consensus with a third rater.

### 2.6 Data Extraction and Analysis

Data extraction of studies meeting inclusion criteria utilized a standardized template developed by the research team. Extracted data focused on public health campaign characteristics (e.g., country, duration, target audiences, strategies deployed, campaign messaging, study design, comparison condition, sample size, outcomes, and results). The full list of data extraction categories can be found in the supplement. Data extraction was conducted independently by two authors; discrepancies were resolved via consensus with a third author. Consistent with PRISMA reporting guidelines, we provide a synthesis of relevant data and a narrative summary of the evidence.

## 3 Results

### 3.1 Search Results

**FIGURE 1:**
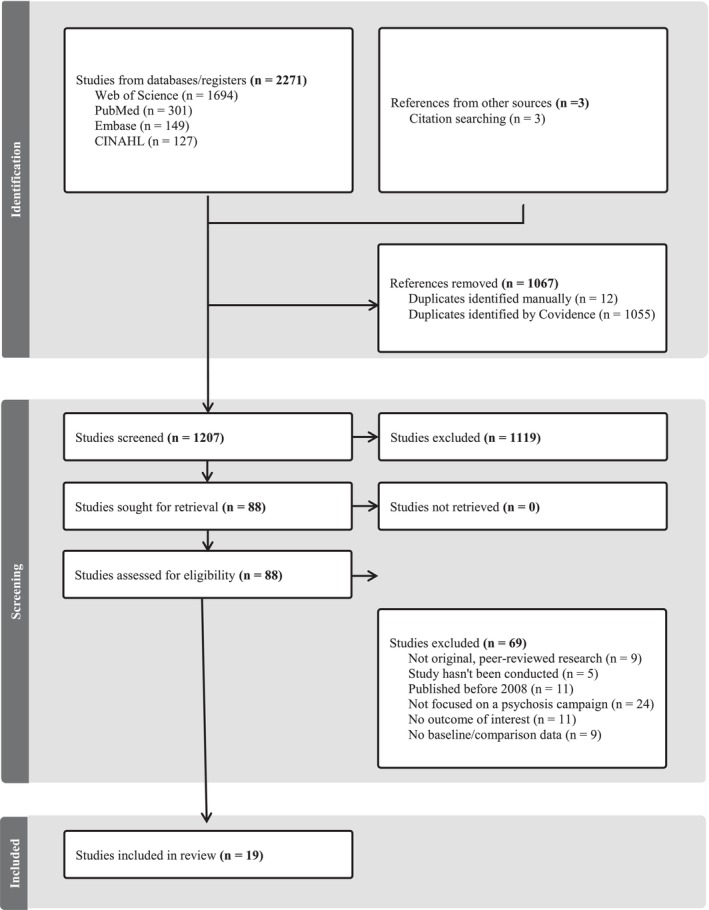
PRISMA consort diagram.

As outlined in Figure 1, database searches initially yielded a total of 2271 articles, of which 1207 qualified for title and abstract screening. After removing articles deemed irrelevant during the title and abstract screening (*n* = 1119) and those that did not meet eligibility criteria upon full text review (*n* = 69), there were 19 articles remaining that qualified for data extraction.

### 3.2 Campaigns

Our final sample consisted of 19 studies describing 14 unique mental health campaigns (see Table 1 for names and characteristics). Four campaigns were conducted in the United States: *Clear Answers to Louisiana Mental Health* (CALM; Weiss et al. 2025); *LaClave* (López et al. 2022), *Mindmap* (Srihari et al. 2022), and *NyWell* (Birnbaum et al. 2024). Four campaigns were conducted in the United Kingdom: *Camden and Islington Early Intervention Service* (CIEIS; Lloyd‐Evans et al. 2015), *Liaison and Education in General Practices* (*LEGs*; Perez et al. 2015), *BiRmingham Early Detection In untREated PsyChosis Trial* (*REDIRECT*; Lester et al. 2009), and *YouthSpace* (Connor et al. 2016). Two campaigns were conducted in Canada: *Prevention and Early Intervention in Psychosis Program* (*PEPP*)*—Montreal* (Jordan et al. 2018; Malla et al. 2014; McIlwaine et al. 2019) and *PEPP—Ontario* campaign (Cassidy et al. 2008). One campaign was conducted in each of the following countries: *Dublin East Treatment and Early Care Team* (*DETECT*; O'Donoghue et al. 2014) in Ireland, *Early Assessment Service for Young People with Psychosis* (*EASY*; Chan et al. 2018) in Hong Kong, *Cologne Early Detection & Therapy Center for Mental Crises* (*FETZ*; Ferrara et al. 2019) in Germany, and *Treatment and Intervention in Psychosis* (*TIPS*) in Norway (Ferrara et al. 2019; Joa et al. 2008; Larsen et al. 2011; ten Velden Hegelstad et al. 2014).

**TABLE 1:** Campaign characteristics.

Campaign	Citation	Country	Duration	Strategies	Messaging	Target audiences
CALM	Weiss et al. 2025	United States	12 months	In‐person events (educational talks, tables at local health fairs and fundraisers), physical advertisements (mass transit and billboard ads), digital media (website including early detection screener (based on PRIME) for individuals and for family, friends, and allies, and social media and digital ads)	Messaging was direct and aimed to convey hope and urgency (“Psychosis is real, so is recovery”. “Losing touch with reality? Let's figure it out”) Information on signs and symptoms of psychosis and how to make a referral to EIS. Relevant sociocultural themes, such as voodoo, were included.	General public
CIEIS	Lloyd‐Evans et al. 2015	United Kingdom	12 months	In‐person events (half‐day workshop to increase awareness of EP and willingness to refer to EIS (included video testimonials from CIEIS clients), monthly meetings with CIEIS worker to discuss concerns or potential referrals, and 1 h review sessions 6–9 months after the initial workshop), physical advertisements (Educational materials were distributed during workshops), digital media (website reinforced campaign messages and provided information for EIS)	Summarized signs of early psychosis and encouraged contacting EIS	Non‐healthcare professionals
DETECT	O'Donoghue et al. 2014	Ireland	78 months^†^	In‐person events (presentations at GP conferences, medical education sessions), physical advertisements (articles in GP newspapers and journals, newsletters, information pack mailed to all GPs in region, articles in local and university newspapers aimed at general public), traditional media (popular soap opera included a storyline about a character with schizophrenia), digital media (website provided information on psychosis and help‐seeking)	Educational program described signs of FEP and benefits of EIS.	General public, healthcare professionals, non‐healthcare professionals
EASY	Chan et al. 2018	Hong Kong	120 months	In‐person events (educational talks and exhibitions to the general public), physical advertisements (posters and leaflets), traditional media (TV advertisements and radio interviews), digital media (campaign website)	Established a new Chinese term for psychosis (old term was translated as “split mind” or “severe mental illness”; new term is translated as “dysregulation in thinking and perception”). Focused on reducing stigma and promoting help‐seeking	General public
FETZ	Ferrara et al. 2019	Germany	36 months	Not reported	Described signs of early psychosis, with a focus on less well‐known, basic, common cognitive‐perceptual symptoms	Healthcare professionals, non‐healthcare professionals
LaCLAve	López et al. 2022	United States	24 months	In‐person events (training events at local churches, community centers, and schools, booths at community events, hosted community roundtable), physical advertisements (bilingual brochures, posters, billboards, etc.), traditional media (advertisements in music, television, and cinema), digital media (campaign website)	All materials in Spanish. Developed the acronym “La Clave” (translated as “the key”) where each letter stands for a symptom of FEP. Tag line “Use la clave to detect serious mental illness”. Creative messaging included music and art with cultural themes.	General public, healthcare professionals, non‐healthcare professionals
LEGs	Perez et al. 2015	United Kingdom	24 months	Low‐ and high‐intensity intervention: physical advertisements (mailed leaflets every 6 months with guidelines for GPs to identify and refer FEP and CHR‐P) High‐intensity intervention only: in‐person events (1‐h educational session (included DVD and PowerPoint presentations) provided by an expert practitioner on detecting FEP and CHR‐P, 1 h booster session 1 year later)	Described signs of FEP and benefits of EIS, included example questions for GPs to screen patients for FEP and information on how to refer to EIS	Healthcare professionals
Mindmap	Srihari et al. 2022	United States	48 months	In‐person events (hosted informational dinners and participated in community events followed by visits to workplaces and ongoing phone and email contact), physical advertisements (newspaper, public transit, postcards, billboards), traditional media (TV and cinema ads), digital media (social media ads on multiple platforms (Facebook, Twitter, YouTube, Instagram, Reddit, and Linkedin) targeted specific groups (e.g., college freshmen orientation))	Simple, visually attractive text normalizing psychosis and encouraged help‐seeking for psychosis. Example flyer text: “People with psychosis are 14× more likely to be the victim of violence than to commit it. Psychosis is not psycho.”, “Something is wrong with your child. Be the first to get them help”, “You wouldn't ignore other parts of your body. Don't ignore your brain”. Tag line “Early detection saves minds”	General public, healthcare professionals, non‐healthcare professionals
NYWell	Birnbaum et al. 2024	United States	18 months	Digital media Youth & Ally websites provided an online mental health quiz, information, live or asynchronous chat, and a place to leave contact information or pursue a referral, web and social media advertisements (Google, Facebook, Instagram)	Encouraged help‐seeking and promoted online screener. Some ads described specific signs of early psychosis while others were more generally focused on mental health. Example ad text: “Hearing whispers—feeling followed—connect with a peer—mental health quiz”, “Free support for families—Understanding the early signs—free wellness assessment—mental health quiz”, “Is it time to check in on your mental health? Free, confidential therapist and peer support in New York—Learn more”	General public
PEPP—Montreal	Jordan et al. 2018; Malla et al. 2014; McIlwaine et al. 2019	Canada	36 months	In‐person events (Educational outreach sessions lasted 60–90 min with booster sessions every 6 months. Sessions included 5‐10 min films (in English and French) with case examples to facilitate case discussions)	Described early signs of FEP and benefits of EIS	Healthcare professionals, non‐healthcare professionals
PEPP—Ontario	Cassidy et al. 2008	Canada	25 months	In‐person events (Held fund‐raising events in partnership with a family support group formed by parents of PEPP), physical advertisements (40 000 colourful posters in key areas such as schools, doctors' offices, shopping malls, and public transit. Distributed pamphlets, calendars, and bookmarks in schools, doctors' offices, and community events), traditional media (short film clip on TV and radio)	Described early signs of FEP and benefits of EIS	General public, healthcare professionals, non‐healthcare professionals
REDIRECT	Lester et al. 2009	United Kingdom	34	In‐person events (educational intervention addressing GP knowledge, skills, and attitudes about FEP). Training included a 17‐min video depicting role‐played primary care consultations with young people with FEP followed by a 15‐min question and answer session including referral guidelines to EIS. (One‐time event with refresher courses during each wave for those trained in earlier waves)	Described early signs of, and encouraged positive attitudes towards, FEP	Healthcare professionals
TIPS	Ferrara et al. 2019; Joa et al. 2008; Larsen et al. 2011; ten Velden Hegelstad et al. 2014	Norway	TIPS 1: 48 months TIPS 2: 30 months TIPS 3: 12 months TIPS 4: 12 months	TIPS 1: In‐person events (educational programs including lectures and videos provided annually to healthcare professionals and education staff), physical advertisements (newspapers ads, posters, mailed brochures to all households in the county), traditional media (commercials shown at the cinema and on local tv and radio stations), digital media (website designed for other professionals to get more information) TIPS 2: No campaign activities except for text‐based advertisements in local paper on an irregular basis TIPS 3: Re‐implemented campaign activities from TIPS 1 TIPS 4: Continued campaign activities from TIPS 3 now including information on substance‐induced psychosis	Described signs of FEP and benefits of EIS, promoted help‐seeking through early detection teams. Example ad text “Mental illness is like any other illness—when you feel something is wrong, you see the doctor”. Slogan “Seek help as early as possible and you have the best chance to recover”	General public, healthcare professionals, non‐healthcare professionals
YouthSpace	Connor et al. 2016	United Kingdom	30 months	In‐person events (psychosis awareness training events were delivered to emergency services, youth and community groups, and employment and education agencies, EIS staff attended community, educational, and NHS events), physical advertisements (Posters displayed in public places and public transit, newspaper and magazine ads, distributed leaflets and postcards), traditional media (radio appearances, short films), and digital media (website was launched for publicity and central information).	Described signs of FEP and encouraged help‐seeking through YouthSpace website or informational line. Example flyer text “Paranoid, Hearing voices, isolated, change of mood—Don't turn your back on the symptoms of psychosis” aimed to raise awareness of signs of EP and how to seek help	General public, healthcare professionals, non‐healthcare professionals

^†^ DETECT did not report an end date for campaign activities. Seventy‐eight months is a minimum estimate of campaign duration based upon reported dates for participant recruitment.

### 3.3 Campaign Duration

Campaign duration ranged from 12 to 120 months with a mean duration of 37.9 months. Table 1 provides the overall duration of each campaign; however, some campaigns did not have consistent activity throughout the duration of the campaign period. For example, the 40‐month PEPP—Montreal campaign included a 6‐month intervention phase, during which events were hosted providing information about FEP and EIS to potential referrers, and a 34‐month post‐intervention phase, during which booster sessions were held every 6 months. Nonconsecutive waves of the TIPS campaign were treated as separate campaigns, such that TIPS 1 had a duration of 48 months and TIPS 3 and 4 (combined) had a duration of 24 months. Additionally, not all campaign strategies were deployed throughout the full duration of the campaigns. For example, as a part of the DETECT campaign, a popular Irish soap opera included a storyline in which an established character developed schizophrenia; this storyline aired for several months early in the campaign and was not repeated.

### 3.4 Target Audiences

Target audiences broadly fell into three categories: healthcare professionals, non‐healthcare professionals, and the general public. Six campaigns (42.9%) targeted the general public as well as healthcare and/or non‐healthcare professionals (DETECT, LaCLAve, Mindmap, PEPP—Ontario, TIPS, and YouthSpace). Three campaigns (21.5%) solely targeted the general public (CALM, EASY, and NYWell). Two campaigns (12.5%) targeted healthcare professionals (LEGs and REDIRECT). One campaign (7.1%), CIEIS, solely targeted staff from non‐healthcare community organizations. Two campaigns (12.5%) targeted both healthcare professionals and non‐healthcare professionals (FETZ and PEPP—Montreal).

### 3.5 Campaign Strategies

Campaign strategies broadly fell into four categories: (1) in‐person events (e.g., hosting educational workshops/trainings and attending community events); (2) physical advertisements (e.g., mailing informational materials, displaying posters and flyers in public places, distributing pamphlets, brochures, etc.); (3) traditional media (e.g., TV, radio, or cinema); and (4) digital media (e.g., campaign website, digital advertising, social media). Three campaigns used a single strategy: PEPP—Montreal and REDIRECT used only in‐person events; NYWell used only digital media. Other campaigns used a combination of strategies, with six campaigns (DETECT, EASY, LaCLAve, Mindmap, TIPS, and YouthSpace) using all four strategies. One campaign (FETZ) did not report campaign strategies.

#### 3.5.1 In‐Person Events

Twelve campaigns (85.7%) hosted in‐person events (CALM, CIEIS, DETECT, EASY, LaCLAve, LEGs, Mindmap, PEPP—Montreal, PEPP—Ontario, REDIRECT, TIPS, and YouthSpace). One popular strategy was hosting educational workshops/trainings for potential referrers (e.g., healthcare professionals, education staff, and/or community workers) aiming to decrease stigma, improve recognition of the signs of early psychosis, and/or promote referral to EIS. Another strategy was to set up a booth or kiosk in public areas (schools, doctor's offices, grocery stores) or at community events to distribute information and create opportunities for community residents to ask questions.

#### 3.5.2 Physical Advertisements

Ten campaigns (71.4%) utilized physical advertisements (CALM, CIEIS, DETECT, EASY, LaCLAve, LEGs, Mindmap, PEPP—Ontario, TIPS, and YouthSpace). Some campaigns mailed information (e.g., pamphlets, brochures, informational packets) to all residents within a region, while others targeted specific audiences, such as healthcare professionals. Campaigns that used this strategy also displayed information in public places, such as advertising on billboards or placing posters and flyers in high‐traffic areas. Campaigns also deployed advertisements in publications such as newspapers, magazines, and medical journals and at in‐person events, as described above.

#### 3.5.3 Traditional Media

Seven campaigns (50%) utilized traditional media (DETECT, EASY, LaCLAve, Mindmap, PEPP—Ontario, TIPS, and YouthSpace). One strategy was to purchase advertisements on TV, radio, or cinema. Another strategy was to feature content within these programs, such as having EIS staff make appearances on a radio show to answer questions or incorporating storylines about psychosis in a television show.

#### 3.5.4 Digital Media

Nine campaigns (64.3%) utilized digital media: seven launched a campaign website (CALM, CIEIS, DETECT, EASY, LaCLAve, TIPS, and YouthSpace). Only two campaigns included digital media beyond a campaign website (Mindmap and NYWell). NYWell. Both Mindmap and NYWell advertised on multiple social media platforms using both text and video ads which were targeted at specific groups based on criteria such as location, demographics, and “interests groups” (e.g., college students, parents, and mental health professionals). Additionally, NYWell placed advertisements on Google; Google search ads were text‐only and included a headline, brief description, and “call to action” (e.g., linking to a free, online mental health quiz and providing a phone number).

### 3.6 Campaign Development

Articles varied greatly in the amount of detail provided regarding campaign development, precluding precise estimates of the frequency with which specific strategies were deployed. Nevertheless, common themes emerged. Early campaign development strategies included referencing previously published work on similar initiatives. For example, the authors associated with the CIEIS campaign conducted and published a systematic review of initiatives to reduce DUP, which concluded that interventions with healthcare professionals failed to reduce DUP (Lloyd‐Evans et al. 2011); this informed the decision for the CIEIS campaign to target only non‐healthcare community staff (Lloyd‐Evans et al. 2015). In addition to referencing published literature, many campaigns described conducting pilot research in their local region, such as conducting focus groups of EIS staff, current or former EIS clients, and their families (Cassidy et al. 2008; Lloyd‐Evans et al. 2015; López et al. 2022; Malla et al. 2014). Furthermore, some campaigns continuously evaluated and adapted campaign tactics throughout the campaign duration (Connor et al. 2016; Srihari et al. 2014). Many campaigns acknowledged the importance of collaborating with stakeholders, particularly those with lived experience, at every stage possible (Birnbaum et al. 2024; Connor et al. 2016; Srihari et al. 2014). For example, the developers of YouthSpace created a Youthboard of young people who had experience in the mental health system and served as advisors throughout the campaign (Connor et al. 2016). Several campaigns also partnered with marketing, media, and/or public relations firms (Birnbaum et al. 2024; López et al. 2022; Srihari et al. 2014; Weiss et al. 2025).

### 3.7 Campaign Messaging

Common themes of campaign messaging included providing information about early signs of psychosis, promoting a non‐stigmatizing attitude towards psychosis, and encouraging help‐seeking. Messaging containing educational information about early signs of psychosis ranged from those focusing on specific symptoms to those providing a broad definition of psychosis. Examples of more specific symptoms include “Hearing whispers—feeling followed—connect with a peer—mental health quiz” (NYWell); “Paranoid, hearing voices, isolated, change of mood—Don't turn your back on the symptoms of psychosis” (YouthSpace); and LaClave, which is an acronym for symptoms of psychosis in Spanish: “Creencias falsas (false beliefs), lenguaje desorganizado (disorganized speech), alucinaciones (hallucinations), ver (seeing [things]), escuchar (hearing [things])”. Examples of broader messaging include CALM's “Losing touch with reality? Let's figure it out” and EASY, which established a new term for psychosis meaning “dysregulation in thinking and perception”. Examples of messages promoting a non‐stigmatizing attitude towards psychosis include “People with psychosis are 14× more likely to be the victim of violence than to commit it. Psychosis is not psycho.” (Mindmap); and “Mental illness is like any other illness—when you feel something is wrong, you see the doctor” (TIPS). Examples of messaging encouraging help‐seeking include “Is it time to check in on your mental health? Free, confidential therapist and peer support in New York—Learn more” (NYWell); “Seek help as early as possible and you have the best chance to recover” (TIPS); and “You wouldn't ignore other parts of your body. Don't ignore your brain—Early detection saves minds” (Mindmap). Cultural adaptations were noted by several campaigns. For example, EASY established a new Chinese term for psychosis in response to the stigma associated with the previous term. LaClave included music and art with cultural themes. CALM incorporated relevant sociocultural themes, such as voodoo, into their messaging.

### 3.8 Outcomes

#### 3.8.1 Dup

**FIGURE 2:**
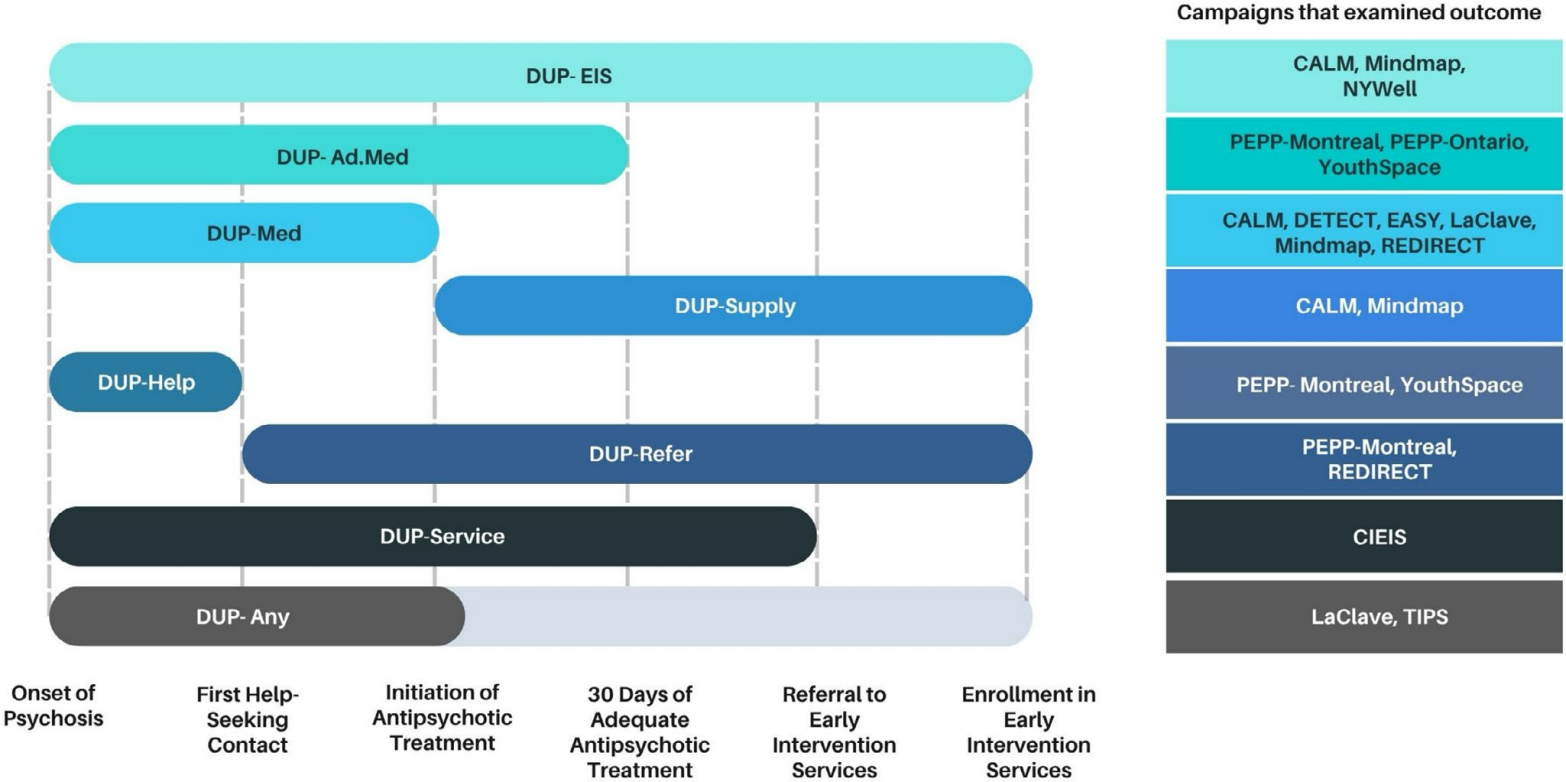
Operational definitions of DUP and DUP subcomponents.

As shown in Table 2, 12 campaigns (85.7%) evaluated the effect of a mental health campaign on DUP (CALM, CIEIS, DETECT, EASY, LaCLAve, Mindmap, NYWell, PEPP—Montreal, PEPP—Ontario, REDIRECT, TIPS, and YouthSpace). Notably, there was significant heterogeneity in the ways that campaigns defined and operationalized DUP, making comparisons across campaigns challenging. While all campaigns used the general definition of DUP as the duration of time from psychosis onset to initiation of treatment, campaigns assessed psychosis onset and treatment initiation differently. Onset of psychosis was defined across studies to correspond to DSM‐5 criteria as assessed with a standardized psychodiagnostic tool administered by a trained rater [e.g., Positive and Negative Syndrome Scale (PANSS), Circumstances of Onset and Relapse Schedule (CORS), Interview for the Retrospective Assessment of the Onset of Schizophrenia (IRAOS), Beiser scale, Nottingham Onset Schedule, and the Structured Interview for Psychosis‐risk Syndromes (SIPS)]. In contrast, the definition of DUP's endpoint (initiation of treatment) varied greatly across studies. As depicted in Figure 2, studies operationalized the conclusion of DUP as initiation of antipsychotic medication treatment, 30 consecutive days of antipsychotic treatment (unless positive symptoms remit sooner), enrollment in EIS, or any of the above. Additionally, some campaigns measured multiple subcomponents of DUP, such as measuring time from psychosis onset to initiation of medication treatment (DUP‐Med) and time from initiation of medication treatment to enrollment in EIS (DUP‐Supply). Campaigns also varied in study design and how DUP outcomes were analysed. Studies compared DUP in the campaign catchment region during the campaign to either DUP in the same region before or after the campaign, or DUP in a comparable geographic region with no campaign; some (e.g., MindMap) used both. Because distribution of DUP tends to be positively skewed, most studies either applied a transformation to DUP data (e.g., log‐transformed or square root‐transformed) or used non‐parametric tests.

**TABLE 2:** Campaign effect on DUP and DUP subcomponents.

Campaign	Design	Sample size	Outcome	Control DUP mean/median (SD^†^)	Campaign DUP mean/median (SD)	Statistical test: significance	Results
CALM	Historical control	Pre‐campaign: *N* = 87 Campaign: *N* = 29	DUP‐EIS	Not reported/26.4 (Not reported)	Not reported/8.4 (Not reported)	χ2: *p* = 0.038	Reduced
			DUP‐Med	Not reported/8.0 (Not reported)	Not reported/0.8 (Not reported)	χ2: *p* = 0.013	Reduced
			DUP‐Supply	Not reported/4.0 (Not reported)	Not reported/4.0 (Not reported)	Not reported, not significant	Unchanged
CIEIS	Historical control	Pre‐campaign: *N* = 70 Campaign: *N* = 110	DUP‐Service	42.1/19.1 (66.9)	56.6/16.6 (106.1)	Mann–Whitney U: *p* = 0.715 Independent samples median test: *p* = 0.875	Unchanged
DETECT	Historical control	Pre‐campaign: *N* = 151 Campaign: *N* = 172	DUP‐Med	Not reported	Not reported	χ^2^: *p* = 0.29	Unchanged
EASY	Historical control examined DUP among adults and young adults (18 to 25 years old)	Adult pre‐campaign: *N* = 88 Adult campaign: *N* = 353	DUP‐Med (Adult)	88.9/25.7 (146.1)	74.8/13.3 (157.1)	Mann–Whitney U: *p* = 0.01	Reduced
		Young adult pre‐campaign: *N* = 34 Young adult campaign: *N* = 126	DUP‐Med (young adult)	Not reported/17.1 (Not reported)	Not reported/12.9 (Not reported)	Mann–Whitney U: *p* = 0.63	Unchanged
LaCLAve	Historical control	*N* = 132	DUP‐Any	Not reported	Not reported	Square‐ root‐transformed data ANOVA: *p* = 0.13	Unchanged
			DUP‐Med	Not reported	Not reported	Square‐ root‐transformed data ANOVA: *p* = 0.43	Unchanged
Mindmap	Historical and comparable region control	Control region baseline: *N* = 12 Control region no‐campaign: *N* = 63 Campaign region baseline: *N* = 24 Campaign region campaign: *N* = 147	DUP‐EIS	Control region baseline: 54.9/46.4 (36.5) Control region no‐campaign: 69.1/61.4 (49.5) Campaign region baseline: 46.6/44.5 (43.3)	Campaign region campaign: 40.6/21.3 (43.1)	Log‐transformed data ANOVA: *p* = 0.39 Quantile regression: Q1, *p* < 0.05 Q2, *p* < 0.0001 Q3, *p* = 0.16	No main effect. Reduced in quartiles 1 and 2, but not 3.
			DUP‐Med	Control region baseline: 29.1/18.1 (30.2) Control region no‐campaign: 25.8/11.6 (33.7) Campaign region baseline: 24.8/14.1 (25.3)	Campaign region campaign: 20.7/6.8 (33.4)	Log‐transformed data ANOVA: *p* = 0.6 Quantile regression: Q1, *p* = 0.25 Q2, *p* = 0.28 Q3, *p* = 0.006	No main effect. Reduced in quartile 3 but not 1 or 2.
			DUP‐Supply	Control region baseline: 25.8/21.7 (25.1) Control region no‐campaign: 42.5/21.3 (44.7) Campaign region baseline: 21.9/4.2 (31.2)	Campaign region campaign: 19.8/2.9 (34.6)	Log‐transformed data ANOVA: *p* = 0.23 Quantile regression: Q1, *p* = 0.39 Q2, *p* = 0.03 Q3, *p* = 0.008	No main effect. Reduced in quartile 3 but not 1 or 2.
NYWell	Stepped‐wedge (clusters of clinics randomized to begin campaign at different times)	Not reported	DUP‐EIS	29.2/Not reported (Not reported)	28.0/Not reported (Not reported)	Log‐transformed data generalized estimating equations: *p* = 0.7	Unchanged
PEPP—Montreal	Historical control	Pre‐campaign: *N* = 136 Campaign: *N* = 159	DUP‐Ad.Med	43.5/17.7 (61.7)	43.5/15.6 (71.6)	Log‐transformed data ANOVA: *p* = 0.712	Unchanged
			DUP‐Help	21.0/6.5 (37.9)	26.3/7.1 (53.4)	Log‐transformed data ANOVA: *p* = 0.481	Unchanged
			DUP‐Refer	13.3/1.2 (35.8)	13.0/1.0 (34.4)	Log‐transformed data ANOVA: *p* = 0.928	Unchanged
PEPP—ontario	Historical control including pre‐campaign, campaign, and post‐campaign timepoints	Pre‐campaign: *N* = 88 Campaign: *N* = 99 Post‐campaign: *N* = 106	DUP‐Ad.Med	Pre‐campaign: Not reported/22.8 (Not reported) Post‐campaign: Not reported/24.5 (Not reported)	Campaign: Not reported/32.5 (Not reported)	Not reported, not significant	Unchanged
RE‐DIRECT	Stratified cluster (primary care practices randomized to receive intervention or control)	Control: *N* = 36 Campaign: *N* = 47	DUP‐Med	33.5/10.1 (41.4)	35.3/8.1 (64.8)	Random effects mixed model: *p* = 0.21	Unchanged
			DUP‐Refer	52.4/25.9 (55.5)	20.4/8.7 (27.5)	Random effects mixed model: *p* = 0.002	Reduced
TIPS^‡^	Naturalistic longitude‐inal design compared across phases of campaign: TIPS Pilot (No campaign), TIPS 1 (campaign) TIPS 2 (no campaign) TIPS 3 (campaign) TIPS 4 (campaign)	TIPS pilot: *N* = 44 TIPS 1: *N* = 146 TIPS 2: *N* = 115 TIPS 3: *N* = 95 TIPS 4: *N* = 108	DUP‐Any	TIPS Pilot: Not reported/26 (Not reported) TIPS 2: Not reported/15 (Not reported)	TIPS 1 (1997–1998): Not reported/6 (Not reported) TIPS 1 (1999–2000): Not reported/14 (Not reported) TIPS 3: Not reported/14 (Not reported) TIPS 4 (2007–2008): Not reported/25 (Not reported) TIPS 4 (2008–2010): Not reported/8 (Not reported)	Log‐transformed data ANOVA: *p* < 0.001	Reduced
youthSpace	Historical and comparable region control	Control region baseline: *N* = 98 Control region no‐campaign: *N* = 74 Campaign region baseline: *N* = 80 Campaign region campaign: *N* = 77	DUP‐Ad.Med	Control region baseline: 30.4/4.5 (65.5) Control region no‐campaign: 30.9/11.4 (47.9) Campaign region baseline: 40.6/10.1 (68.9)	Campaign region campaign: 14.8/5.6 (22.1)	Log‐transformed data mixed model ANOVA: *p* = 0.0039	Reduced

^†^ SD: standard deviation.

^‡^ Table reports TIPS DUP outcomes described in Hegelstad et al. 2014. Additional articles report additional analyses related to specific waves of TIPS, while Hegelstad et al. 2014 reports DUP results across all four waves of the TIPS campaign.

Six campaigns (50%) did not detect a significant effect on DUP (CIEIS, DETECT, LaClave, NYWell, PEPP—Montreal, and PEPP—Ontario). Four campaigns (33%) did detect a statistically significant reduction on overall DUP (CALM, EASY, TIPS, and YouthSpace). CALM reduced DUP‐EIS and DUP‐Med but not DUP‐Supply; EASY reduced DUP‐Med for adults but not young adults (18–25 years old); TIPS reduced DUP‐Any; YouthSpace reduced DUP‐Ad.Med. Furthermore, two additional campaigns, which did not result in a reduction in overall DUP, did show a reduction when using advanced statistical techniques to parse DUP or when examining a specific subcomponent of DUP (Mindmap and REDIRECT). Mindmap was found to have no effect on DUP‐EIS, DUP‐Med, or DUP‐Supply when using mean comparison analyses. However, subsequent quantile regression revealed that DUP‐EIS was reduced in the first and second quartile but not the third; DUP‐Med and DUP‐Supply were both reduced in the third quartile but not the first or second. REDIRECT was found to have no effect on DUP‐Med but reduced DUP‐Refer.

#### 3.8.2 Referrals and Enrollment

As shown in Table 3, five campaigns (35.7%) evaluated referral and enrollment metrics (FETZ, LEGs, NYWell, PEPP—Montreal, and REDIRECT). FETZ examined initial contact with the EIS, regardless of diagnosis or eventual enrollment status, and found that referrals increased following the launch of the campaign (test‐statistic and significance not reported). NYWell examined both referrals and new enrollment and concluded that neither was affected by the campaign (test‐statistic and significance not reported). REDIRECT examined referrals who met criteria for a first episode of psychosis (FEP) and found no difference in the number of FEP cases referred by primary care practices that had been randomized to receive a high‐intensity intervention compared to practices that received a low‐intensity intervention. PEPP—Montreal examined FEP cases, clinical high risk for psychosis (CHR‐P) referrals, and proportion of non‐cases (referrals who did not meet criteria for FEP or CHR‐P) and found that all increased during the campaign. LEGs examined true‐positives (referrals who met criteria for FEP or CHR‐P) and false‐positives (referrals who did not meet criteria for FEP or CHR‐P) and found that primary care practices receiving a high‐intensity intervention referred more often, yielding both more true‐ and false‐positives, compared to practices receiving a low‐intensity intervention or continuing practice as usual.

**TABLE 3:** Campaign effect on referrals and enrollment.

Campaign	Design	Sample size	Outcome	Control outcome	Campaign outcome	Statistical test: significance	Results
FETZ	Historical control tracked referrals to FETZ before and during the campaign	N/A	Referrals—Initial contacts made to FETZ each year	Year 1: 50 Year 2: 69 Year 3: 113	Year 1: 198 Year 2: 233 Year 3: 209	Not reported	Reported increase but statistical significance not reported
LEGs	Compared referrals to EIS made by primary care practices randomized to the high‐intensity intervention to those randomized to the low‐intensity intervention (described in Table 1) and those continuing practice‐as‐usual (PAU)	High‐intensity: *N* = 24 Low‐intensity: *N* = 28 PAU: *N* = 50	True‐positives: Number of referrals to EIS made by each primary care practice who met criteria for FEP or CHR‐P	PAU: 30 Low‐Intensity: 32	High‐intensity: 53	High vs. low, Incident rate ratio (IRR): *p* = 0.02 High vs. PAU, IRR: *p* < 0.0001 Low vs. PAU, IRR: *p* = 0.1	More true positives referred from high‐intensity practices compared to low‐intensity or PAU (no difference between low‐intensity and PAU)
			False‐positives: Number of referrals to EIS made by each primary care practice, who did not meet criteria for FEP or CHR‐P	PAU: 38 Low‐Intensity: 25	High‐intensity: 56	High vs. low, Incident rate ratio (IRR): *p* = 0.005 High vs. PAU, IRR: *p* < 0.0001 Low vs. PAU, IRR: *p* = 1.0	More false positives referred from high‐intensity practices compared to low‐intensity or PAU (no difference between low‐intensity and PAU)
NYWell	Stepped‐wedge (clusters of clinics randomized to begin campaign at different times)	*N* = 6 clusters of FEP clinics were randomized within the stepped‐wedge design and experienced a baseline and active campaign period.	Referrals—Mean number referrals made to OTNY	*M* = 89.88 (SD = 8.86)	*M* = 86.29 (SD = 26.10)	Not reported	Unchanged. Statistical significance not reported
			New admissions—mean number of individuals who met criteria for, and enrolled in services at, OTNY	*M* = 29.07 (SD = 2.22)	*M* = 32.10 (SD = 5.38)	Not reported	Unchanged. Statistical significance not reported
PEPP‐montreal	Historical Control	N/A	FEP Cases—Number of referrals who met criteria for FEP	136	159	Not reported	Reported increase but statistical significance not reported
		N/A	CHR‐P Referrals—Mean number of referrals to CHR‐P program every 6 months (Screened by FEP program and sent to CHR‐P if deemed appropriate)	*M* = *7*.*67* (SD = 0.58)	*M* = 1*7*.*67* (SD = *6*.71)	T‐test: *p* = 0.01	Increased
		N/A	Proportion of non‐cases—Proportion of referrals to who did not meet criteria for FEP	198/335 (59%)	*N* = 307/463 (66%)	Z‐test: *p* = 0.02	Increased
REDIRECT	Stratified cluster (primary care practices randomized to receive intervention or control)	N/A	FEP Cases: Number of referrals who met criteria for FEP	82	97	Non‐linear mixed model: *p* = 0.48	Unchanged

## 4 Discussion

This scoping review of the international literature on public health campaigns intended to support early detection of psychotic disorders yielded a total of 19 studies published between February 2008 and June 2025. Studies reported DUP and/or DUP proxy outcomes associated with 14 distinct campaigns. Not surprisingly, campaigns utilized a range of strategies, messaging frameworks, and target audiences. Twelve campaigns reported DUP. In addition to, or in place of, measuring DUP, five campaigns measured referrals and enrollment metrics. Notably, there was significant heterogeneity in how DUP was operationalized across studies. Inconsistent definitions impeded cross‐campaign comparisons and evidence synthesis. Thus, we found it necessary to attend to the specific operational definitions of DUP used by each campaign to account for potential variability in DUP estimates (Figure 2). This observation is not unique to our analysis. Indeed, the empirical literature frequently critiques the DUP construct due to several methodological and conceptual challenges that hinder comparability, replication, and interpretation of research findings (Polari et al. 2011; Register‐Brown and Hong 2014). We address this limitation for the field in our recommendations below. We found mixed evidence of campaign effectiveness for reducing DUP and referral/enrollment metrics. Many studies detected changes in DUP subcomponents but not in the broader construct of DUP, which suggests that interventions may be influencing specific stages of the help‐seeking and treatment pathway (e.g., earlier recognition or faster referral) without yet producing sufficient cumulative impact to shorten the overall duration from symptoms onset to treatment initiation. Similarly, campaigns like MindMap and REDIRECT, which showed no overall effect on DUP using mean comparisons, demonstrated DUP reductions in specific DUP subcomponents or when analysed with advanced methods (e.g., quantile regression), which also suggests that meaningful changes may be obscured in traditional average‐based analyses.

Campaigns varied widely in their audiences, with some targeting only the general public or specific professionals, and others combining both. Unsurprisingly, campaigns targeting only (healthcare and/or non‐healthcare) professionals were unlikely to reduce DUP‐Help or overall DUP; some did show reduction in DUP‐Refer. This suggests that past research, which concluded that interventions with healthcare professionals were ineffective at reducing overall DUP (Lloyd‐Evans et al. 2011), may fail to account for important effects on DUP subcomponents. Campaigns that targeted multiple groups (e.g., both the public and professionals) were relatively common and may have a broader impact, but they necessitate audience‐specific strategies to ensure relevance and effectiveness.

Given that the timeframe for our literature review coincided with a digital growth spurt likened to the industrial revolution (Guo et al. 2020; Wang et al. 2023), it was not surprising that more recent campaigns were more likely to deploy messaging through digital media channels. The majority of campaigns launched a website as their sole digital campaign strategy. Only two of the most recent campaigns (Mindmap and NYWell) utilized additional aspects of digital media such as social media advertising. The effectiveness of digital advertising remains unclear as Mindmap achieved reductions in aspects of DUP while NYWell did not. Notably, NYWell comprised only digital media while Mindmap used a variety of strategies including in‐person events, physical advertisements, and traditional media in addition to digital media. Thus, preliminary conclusions suggest that digital media may be effective as a part of a multi‐component campaign but is likely insufficient to reduce DUP as a sole strategy.

Similarly, utilizing multiple diverse campaign strategies was associated with a higher likelihood of achieving the primary campaign objective. For instance, no campaigns deploying a single strategy (NyWell, PEPP—Montreal, and REDIRECT) reduced overall DUP. However, as discussed above, REDIRECT, which comprised only in‐person events for healthcare professionals, was found to reduce DUP‐Refer. It is likely that more limited campaigns, while potentially sufficient to educate potential referrers and reduce DUP‐Refer, are insufficient to impact the general public and reduce DUP‐Help or overall DUP. Other campaigns comprised three or four strategies (in‐person events, physical advertisements, and one or both of traditional and/or digital media). CALM, EASY, and Mindmap were found to reduce aspects of DUP, but not overall DUP. TIPS and YouthSpace resulted in significant reductions in overall DUP. Relatedly, campaign intensity may influence referral volume and quality. The LEGs campaign demonstrated that a high‐intensity intervention increased both true‐ and false‐positive referrals, suggesting that while awareness efforts can boost overall referral rates, they may also lead to more inappropriate referrals without sufficient targeting.

### 4.1 Recommendations

Based on the findings of this scoping review, the following recommendations outline key strategies for designing and deploying public health campaigns aimed at effectively signposting the public to EIS.

#### 4.1.1 Refine and Standardize Definitions of DUP

We echo previous calls for a consensus definition of DUP and standardized assessment tools that can enhance consistency and comparisons. Much of the existing evidence linking DUP to clinical outcomes has operationalized DUP as time to initiation of antipsychotic medication or entry into services where such treatment is standard. Given that medications are not a universal component of an individual's treatment plan, and given well‐documented challenges with medication adherence, we advise caution in relying exclusively on psychopharmacologic metrics to define the end of the DUP period. Furthermore, we acknowledge the problems with using enrollment in EIS as an appropriate benchmark, given the access issues for historically underserved populations. We therefore recommend operationalizing the end of the DUP period as the initiation of any treatment for psychotic symptoms, including psychotherapy for psychosis, antipsychotic medication, or early psychosis specialty care. We recognize that this broader definition introduces potential challenges, including variability in what constitutes psychosis‐specific care and limited evidence regarding its association with clinical outcomes. As such, this approach should be considered complementary and hypothesis‐generating, with future research needed to establish its predictive validity. We further argue that DUP as a global construct is inherently limiting given the multitude of factors that are associated with a prolonged DUP, such as stigma, distance from a clinic, and administrative delays. We therefore advocate for a shift from the global DUP construct toward a more specific analysis of DUP subcomponents, as articulated by Malla et al. (2021). Identifying universal and setting‐specific contributors to DUP subcomponents may support more precise measurement, facilitate comparisons across studies, and guide targeted public health, clinical, and policy interventions. For example, the United Kingdom's National Health Service mandates a 2‐week maximum window for treatment initiation for a new onset psychosis (operationalized as time from referral to initiation of treatment). To support this mandate systematically, the NHS England Access and Waiting Time Standard for Early Intervention in Psychosis identified compliance standards across the NHS that increased over time (Adamson et al. 2018). This standard should serve as a key performance indicator for systems internationally and potentially as a more pragmatic outcome variable in research.

#### 4.1.2 Evaluate Proxy Outcomes

While reducing DUP remains a priority, public health interventions, particularly those that leverage a range of strategies, are encouraged to report outcomes that may be mechanistically associated with DUP. Outcomes related to facilitating treatment access, such as EIS referral and enrollment, are not only useful proximal indicators of DUP but also represent meaningful targets in and of themselves; increasing the number of individuals accessing EIS may be an equally valuable outcome as reducing DUP. Furthermore, accounting for referral and/or enrollment may help to elucidate the effect of campaigns on DUP given previous evidence that DUP may increase early in a campaign, presumably due to the detection of individuals with extremely long DUP who likely would not have connected with care otherwise (Friis et al. 2005; Malla et al. 2005).

#### 4.1.3 Develop Standards for Health Campaign Research

The variability in methodologies used to evaluate the effectiveness of psychosis awareness campaigns limits the ability to draw meaningful comparisons or conclusions across studies. Notably, some studies employed contemporaneous control groups, while others relied on historical controls or lacked clear comparison conditions altogether. These design choices introduce distinct vulnerabilities to bias—for instance, historical controls are particularly susceptible to temporal confounding and shifts in service infrastructure or public awareness, whereas contemporaneous controls, while stronger, may still face challenges such as selection bias. As Friis et al. (2012) noted, failure to account for these biases can undermine confidence in observed effects. Future campaign evaluations would benefit from methodological standards that promote the use of rigorous, clearly defined control conditions and that explicitly address potential sources of bias in study design and interpretation.

#### 4.1.4 Leverage Digital Media Strategies

Digital marketing offers greater precision, reach, and interactivity in health communication campaigns compared to traditional strategies. It allows for real‐time data tracking, targeted messaging to specific populations, and two‐way engagement. This makes campaigns more adaptable, responsive, scalable, and—potentially—cost‐effective, and therefore more likely to achieve campaign objectives. Campaigns should leverage social medial platforms like TikTok, Instagram, and YouTube to share short‐form content that can normalize help‐seeking, highlight early signs of psychosis, and feature stories from individuals with lived experience. While not identified as a strategy in the campaigns we reviewed, partnerships with influencers and community leaders can further promote campaign messages and increase credibility and reach. Digital ads can be geo‐targeted toward areas with historically high DUP or poor access to care, providing an illuminated and streamlined pathway to EIS. Search engine optimization (SEO) and paid search ads can ensure that accurate, stigma‐free information about psychosis and early treatment is presented in response to searches about psychotic or psychotic‐like experiences. Mobile‐compatible campaign websites create ease of access to psychosis screening tools, informational resources, and direct referral pathways to early psychosis services. Finally, unlike out‐of‐home placements like billboards and posters, many digital strategies offer engagement metrics (e.g., click‐through rates, views, time on page) that can be monitored in real‐time and used to inform revisions to messages, design, audiences, and other approach‐ and content‐based decisions to maximize return on investment as well as outcomes.

#### 4.1.5 Streamline Access to Care and Educate Gatekeepers

When possible, streamline access to care and remove barriers, such as requiring a referral from a PCP, which can lengthen DUP and disproportionately affect marginalized populations. Allowing for direct referrals from individuals experiencing psychosis and their families allows for a quicker, more direct pathway to care and eliminates opportunities for aversive, stigmatizing encounters. When it is impossible to remove such barriers, educate relevant gatekeepers with targeted information about recognizing the signs of psychosis and referring appropriate individuals to EIS.

#### 4.1.6 Design Multi‐Phase Campaigns With Sustained Engagement and Iteratively Adapt Campaign Messaging and Strategies

Campaign developers should consider structuring efforts in phases—an initial intensive outreach phase followed by periodic reinforcement (e.g., booster sessions)—to maintain momentum and institutional memory, especially in systems with high staff turnover. Furthermore, campaigns should evaluate early results in order to adapt campaign messaging and strategies in an iterative manner informed by recent data from the local region.

#### 4.1.7 Collaborate With Stakeholders During Campaign Development

The lack of consistent reporting on campaign development activities hindered a comprehensive analysis of key processes. We recommend actively involving the target audiences of the campaign in both the development and testing of campaign messaging and branding to ensure that the campaigns are relevant, relatable, and impactful. We also recommend actively involving relevant stakeholder groups, including people with lived experience, family members, and representatives from the EIS in the campaign catchment region to ensure that the campaign goals and strategies are in alignment with their needs and preferences.

#### 4.1.8 Tailor Messaging by Audience Segment

Future campaigns should clearly segment and customize content for different target groups (e.g., youth vs. healthcare professionals) to ensure the messaging addresses each group's unique knowledge gaps, motivations, and roles in reducing DUP and increase the likelihood of engaging messaging. When targeting the general public, it is important for messages to be normalizing, non‐stigmatizing, and promote specific action. When targeting healthcare professionals, provide advice on how to approach conversations about psychosis in a direct and non‐stigmatizing manner.

#### 4.1.9 Enhance Targeted Outreach to Underserved Communities

While culturally adapted campaign messaging is essential, it may not be sufficient on its own to effectively promote help‐seeking behaviors among historically underserved populations. We recommend a multifaceted approach that includes targeted outreach through community events and collaborations with trusted stakeholders who hold credibility and influence within these communities.

## 5 Conclusion

This scoping review underscores both the promise and the persistent challenges of public health campaigns designed to reduce DUP. While a subset of campaigns demonstrated effectiveness—particularly those employing multi‐modal, targeted, and sustained strategies—findings were constrained by inconsistent definitions, methodological heterogeneity, and an overreliance on average‐based analyses that may obscure meaningful subgroup effects. Our synthesis points to several actionable pathways forward: the need for a consensus definition and disaggregation of DUP; the utility of proxy metrics such as referral volume and service engagement; and the strategic potential of digital media to reach high‐risk populations with tailored, stigma‐reducing content. To accelerate progress, future campaigns should emphasize audience‐specific strategies, integrate rigorous evaluation frameworks from implementation science, and leverage multiple strategies including online digital approaches. With greater conceptual precision, technological innovation, and stakeholder engagement, public awareness campaigns can more effectively promote timely access to care and improve trajectories for individuals experiencing or at risk for psychosis.

## Funding

This work was supported by Washington State Health Care Authority, K5559 WO13.

## Conflicts of Interest

The authors declare no conflicts of interest.

## Supporting information


**Data S1:** Preferred Reporting Items for Systematic reviews and Meta‐Analyses extension for Scoping Reviews (PRISMA‐ScR) Checklist (p. 2–3).
**Data S2:** Full search strategy (p. 4).
**Data S3:** Full list of data extraction categories (p. 5).

## Data Availability

The data that support the findings of this study are available from the corresponding author upon reasonable request.
